# Gallic Acid Suppressed Tumorigenesis by an LncRNA MALAT1-Wnt/β-Catenin Axis in Hepatocellular Carcinoma

**DOI:** 10.3389/fphar.2021.708967

**Published:** 2021-10-06

**Authors:** Chuan-jian Shi, Yan-biao Zheng, Fei-fei Pan, Feng-wei Zhang, Peng Zhuang, Wei-ming Fu

**Affiliations:** ^1^ School of Pharmaceutical Sciences, Southern Medical University, Guangzhou, China; ^2^ Guangdong Provincial Key Laboratory of New Drug Screening, School of Pharmaceutical Sciences, Southern Medical University, Guangzhou, China; ^3^ Department of Oncology, The Sixth People’s Hospital of Huizhou, Huiyang Hospital Affiliated to Southern Medical University, Huizhou, China; ^4^ Key Laboratory of Orthopaedics and Traumatology, The First Affiliated Hospital of Guangzhou University of Chinese Medicine, The First Clinical Medical College, Guangzhou University of Chinese Medicine, Guangzhou, China; ^5^ Lingnan Medical Research Center, Guangzhou University of Chinese Medicine, Guangzhou, China; ^6^ The Eighth Affiliated Hospital of Sun Yat-sen University, Shenzhen, China

**Keywords:** hepatocellular carcinoma, gallic acid, lncRNA MALAT1, Wnt/β-catenin signaling, proliferation, metastasis

## Abstract

Gallic acid (3,4,5-trihydroxybenzoic acid; GA), a natural phenolic acid, is abundantly found in numerous natural products. Increasing evidence have demonstrated that GA plays anti-cancer roles in multiple cancers. However, its anti-tumor effects on hepatocellular carcinoma (HCC) and the underlying mechanism remain obscure. In the present study, we found that GA suppressed the *in vitro* cell viability and metastasis and inhibited the *in vivo* tumor growth of HCC cells. The underlying mechanism was further to investigate and it was showed that GA suppressed the expression of β-catenin and led to the functional inactivation of Wnt/β-catenin signaling. As a kind of significant regulators, the long noncoding RNA molecules (lncRNAs) have attracted widespread attentions for their critical roles in diverse biological process and human diseases. To further identify which lncRNA participated this GA-mediated process, several lncRNAs related to Wnt/β-catenin signaling were chosen for examination of their expression profiling in the GA-treated HCC cells. Of which, Metastasis-Associated Lung Adenocarcinoma Transcript 1 (MALAT1) was the most promising candidate. And moreover, MALAT1 was significantly down-regulated by GA. Its overexpression partially reversed the GA-induced the inhibitory effects on cell proliferation and metastasis; and successfully abolished the suppressive effect of GA on Wnt/β-catenin signaling. In conclusion, our results indicated that GA suppressed tumorigenesis *in vitro* and *in vivo* by the MALAT1-Wnt/β-catenin signaling axis, suggesting that GA has great potential to be developed as a chemo-prevention and chemotherapy agent for HCC patients.

## Introduction

Hepatocellular carcinoma (HCC) accounts for the majority of primary liver cancers and it has been considered as the fourth most common cause of cancer-related death worldwide (Villanueva, 2019). Most of patients are diagnosed at an advanced stage and lost the chance of surgery for the lack of effective early diagnostic biomarkers. And combined with the lack of effective therapeutic targets and drugs, these HCC patients often have poorer survival (Klingenberg et al., 2017; Huang et al., 2020). Therefore, looking for effective biomarkers and therapeutic drugs are urgent for the treatment of HCC. Natural products bring a new hope for the discovery of anti-cancer candidates considering its low toxicity and multi-target (Harvey, 2008). A number of plant-derived compounds and their derivatives have been demonstrated to play significant roles in prevention and treatment of cancers (Lin et al., 2015; Xu et al., 2017; Li et al., 2018). Gallic acid (GA), 3,4,5-trihydroxybenzoic acid, is a phenolic acid derived from gallnuts, teas, grapes, red and white wine, sumac and other natural products (Shahrzad et al., 2001; De Beer et al., 2003; Ma et al., 2003). Various biological activities of GA have been documented in previous studies, such as anti-oxidant, anti-inflammation, anti-tumor, anti-mutagenic action, anti-bacteria, neuroprotection and cardiovascular protection (Locatelli et al., 2013; Shao et al., 2015; Choubey et al., 2018; Kahkeshani et al., 2019). Regarding the anti-cancer activity, GA has been reported to exert beneficial effects on various cancers such as leukemia (Madlener et al., 2007), prostate cancer (Chen et al., 2009), lung cancer (Park and Kim, 2013), gastric cancer (Ho et al., 2013), colorectal, breast, cervical and esophageal cancers (Choubey et al., 2018). However, the function of GA in HCC remains unclear, and more investigation are needed to clarify this anti-cancer activity.

Long non-coding RNAs (lncRNAs) have emerged as potential key regulators of cancer biology. Accumulating evidence suggests that aberrant expression of long non-coding RNAs (lncRNAs) play an important functional role in tumorigenesis and progression (Batista and Chang, 2013). Intriguingly, lncRNAs act as HCC diagnosis marker alone or combined with other well known biomarker such as AFP. For instance, two lncRNAs UCA1 and WRAP53 combined with AFP achieved HCC sensitivity up to 100% (Klingenberg et al., 2017; Huang et al., 2020). As a widely studied lncRNA, Metastasis Associated Lung Adenocarcinoma Transcript 1 (MALAT1) has been reported to be frequently up-regulated in tumors, and closely correlate with tumor grades and metastasis in multiple cancers, such as lung cancer, breast cancer, prostate cancer, osteosarcoma, gastric cancer (Schmidt et al., 2011; Chang et al., 2020; Goyal et al., 2021). In HCC, MALAT1 promotes cell proliferation, epithelial-mesenchymal transition (EMT), migration and sorafenib resistance in HCC cells (Fan et al., 2020), suggesting its diagnostic, prognostic, and therapeutic role in HCC. We therefore considered that MALAT1 maybe a promising targets for HCC diagnosis and therapeutic strategy.

In the present study, we systematically examined the anti-cancer effect of GA on HCC, and it was found that GA suppressed the proliferation and metastasis *in vitro* and inhibit tumor growth *in vivo*. Further investigation also showed that GA significantly suppressed the expression of MALAT1, and led to the inactivation of Wnt/β-catenin signaling. Therefore, our data indicated that GA suppressed tumorigenesis by modulating lncRNA MALAT1-Wnt/β-catenin signaling axis in HCC, suggesting that it may be a promising drug candidate for HCC patients.

## Materials and Methods

### Reagents and Antibodies

Gallic acid (purity >98% by HPLC, Cat. No. S30153) was purchased from Shanghai yuanye Bio-Technology (Shanghai, China), and dissolved in PBS for the usage. The primary antibodies were anti-β-catenin (Cat. No. 8084S), Lamin B1 (Cat. No.13435S) and GAPDH (Cat. No.5174S) were purchased from Cell Signaling Technology (Danvers, MA, United States). Anti-E-cadhein (Cat.NO: ab231303), Vimentin (Cat.NO: ab92547) and MMP9 (Cat.NO: ab76003) were purchased from Abcam (CA, United States). The secondary antibody (Cat. No. AP132P) were purchased Merck Millipore (MA, United States).

### Cell Culture

The human HCC cell lines, HepG2, Bel-7402 and the immortalized nontumorigenic cell line LO2, were cultured in Dulbecco’s modified Eagle’s medium (DMEM, Gibco) with 10% fetal bovine serum (FBS, ExCell Bio) and 0.1% penicillin/streptomycin (Invitrogen, Carlsbad, CA, United States). These cells were incubated and maintained in a humidified atmosphere at 37°C, 5% CO_2_.

### Cell Viability Assays

Cells were seeded in 96-well plates at a density of 5 × 10^3^ cells per well and treated with series concentrations of GA (0, 50, 100, 150, 200, 400 μM) for 24, 48 and 72 h. The Cell Counting Assay Kit-8 solution (Beyotime, Shanghai, China) was added and further incubated for another 1 hour at 37°C. The absorbance was measured at 450 nm using a Hybrid Multi-Mode Microplate Reader (Tecan, Switzerland). All the experiments were performed in triplicates.

### Colony Formation Assays

The HepG2 and Bel-7402 cells were treated with 120 μM GA for 48 h in dishes and subsequently re-plated into 6-well plates at the density of 100–200 cells per well and then cultured for 2 weeks to evaluate the clonogenic ability. The colonies were fixed with methanol and stained with Giemsa staining solution for 15 min. The images were captured using the ImmunoSpot analyzer (CTL, United States), and the colony numbers were counted by ImmunoSpot® Version 6.0 Academic system. The plating efficiency (PE) and surviving fraction (SF) were calculated according to previous report (Franken et al., 2006).

### Flow Cytometry Examination

For apoptosis analyses, HCC cells were seeded in 6-well plates and treated with 120 μM GA for 48 h. Then, cells were collected and washed with cold PBS, and followed by annexin V and propidium iodide (PI) incubation according to the protocol of the Apoptosis Detection Kit (KeyGEN, Nanjing, China). The stained cells were monitored by flow cytometry (Beckman, Pasadena, CA). For cell cycle analyses, cells were harvested and stained with the dye from the Cell Cycle Detection Kit (KeyGEN) according to the manufacturer’s protocol. Then, DNA content was subjected to flow cytometry (Beckman) examination. Each assay was conducted in triplicates.

### Wound Healing and Transwell Assays

For wound healing assays, cells were plated into 12-well plates and incubated overnight. To prevent the influence of cell proliferation, cells were pre-treated with mitomycin C (10 μg/ml) for 1 h, and then the monolayer was carefully scratched with a sterile plastic tip, and washed with PBS for three times to remove floating cells. Then the cells were cultured in the presence or absence of GA in serum-free media for 48 h, and images were captured under a microscope. The status of wound area was evaluated by ImageJ. The relative wound area was calculated according to the formula: S_48_/S_0_, where S_0_ and S_48_ are the wound area at 0 and 48 h respectively.

For transwell assays, 5.0 × 10^4^ GA-treated cells and control cells were resuspended in serum-free medium and seeded in the upper chamber, which was coated with Matrigel (Corning, United States). Complete DMEM with 10% FBS was added into the lower chamber. After incubation for 20–36 h, the cells on the lower membrane were fixed with methanol for 20 min and stained with 0.1% crystal violet. Images were captured by Multifunctional Cell Imaging Microplate reader (BioTek, United States) and invaded cells were analyzed by using Gene5 software.

### Cell Transfection

The MALAT1 overexpression plasmid was generated according to previous study and the pcDNA3.1 (+) were used as control (Zhang et al., 2019). The HepG2 cells were seeded in six-well plates. After incubation for 12 h, cells were transfected with pMALAT1 and pcDNA3.1 (+) by using Lipofectamine 3,000 (Invitrogen) respectively.

### RNA Extraction, Reverse Transcription, and Quantitative Real-Time Polymerase Chain Reaction

Total RNA was extracted using Animal Total RNA Isolation Kit (FOREGENE, Chengdu, China), and it was reversely transcribed using PrimeScript RT Reagent Kit (Takara, Japan). The qRT-PCR examinations were conducted using Power up SYBR Green Master Mix (Thermo Fisher Scientific, Waltham, MA) on a Light-Cycler480 System (Roche, Basel, Switzerland). The primer sequences were listed in Table1. GAPDH was served as the endogenous control and fold changes were calculated by means of relative quantification (2^–ΔΔCt^). All the experiments were performed in triplicates.

**TABLE 1:** The primer sequences used in this study.

Gene	Forwardprimer (5–3′)	Reverse primer (5–3′)
GADPH	GCACCACCAACTGCTTAG	TCT​TCT​GGG​TGG​CAG​TGA​TG
Survivin	CCA​CCG​CAT​CTC​TAC​ATT​CAA​G	CAA​GTC​TGG​CTC​GTT​CTC​AGT​G
Cyclin D1	CTG​GAG​GTC​TGC​GAG​GAA​CA	CCT​TCA​TCT​TAG​AGG​CCA​CGA​A
Oct3/4	TCG​AGA​ACC​GAG​TGA​GAG​GC	CAC​ACT​CGG​ACC​ACA​TCC​TTC
VEGF	AGG​GCA​GAA​TCA​TCA​CGA​AGT	AGG​GTC​TCG​ATT​GGA​TGG​CA
E-cadherin	CGA​GAG​CTA​CAC​GTT​CAC​GG	GGG​TGT​CGA​GGG​AAA​AAT​AGG
Vimentin	GAC​GCC​ATC​AAC​ACC​GAG​TT	CTT​TGT​CGT​TGG​TTA​GCT​GGT
N-cadherin	GCC​AGG​ATG​CCG​AAA​ATT​AG	CCA​GCC​TTC​TCG​TCA​AAT​CCT
Fibronectin	CGG​TGG​CTG​TCA​GTC​AAA​G	AAA​CCT​CGG​CTT​CCT​CCA​TAA
ZEB1	ATC​ATC​GCT​ACT​CCT​ACT​G	TCTTCCCTTGTCAAACTC
ZEB2	CAA​GAG​GCG​CAA​ACA​AGC​C	GGT​TGG​CAA​TAC​CGT​CAT​CC
SNAI1	TCG​GAA​GCC​TAA​CTA​CAG​CGA	AGA​TGA​GCA​TTG​GCA​GCG​AG
SNAI2	CGA​ACT​GGA​CAC​ACA​TAC​AGT​G	CTG​AGG​ATC​TCT​GGT​TGT​GGT
TWIST	GGA​GTC​CGC​AGT​CTT​ACG​AG	TCT​GGA​GGA​CCT​GGT​AGA​GG
HOTTIP	CCT​AAA​GCC​ACG​CTT​CTT​TG	TGC​AGG​CTG​GAG​ATC​CTA​CT
H19	TGC​TGC​ACT​TTA​CAA​CCA​CTG	ATG​GTG​TCT​TTG​ATG​TTG​GGC
TINCR	TGT​GGC​CCA​AAC​TCA​GGG​ATA​CAT	AGA​TGA​CAG​TGG​CTG​GAG​TTG​TCA
HULC	TTC​ACC​AGG​CTG​ATA​GTC​CG	ACA​CGT​CCT​TCC​CAT​AAA​CCC
MALAT1	TGG​AAT​TTG​GAG​GGA​TGG​GAG​GAG	ACT​TGC​CAA​CAG​AAC​AGA​CAG​ACC
ROR	TCC​AAC​TCA​CCT​GAC​AGC​CAC​TC	CAG​TCT​TCA​GCC​GCT​AAG​CCA​AG

### Luciferase Activity Assay

The luciferase activity assay was carried out as described previously (Liang et al., 2019). After HCC cells were seeded in 12-well plates, the luciferase reporter TOPflash and pRL-TK plasmids (internal control for normalization) were co-transfected into cells by Lipofectamine 3,000 (Invitrogen). Twelve hours later, cells were treated with GA for 48 h, and cells were lysed and subjected to luciferase activity assays by using the Dual-Luciferase® Reporter Assay System (Promega, Madison, WI, United States) on a Hybrid Multi-Mode Microplate Reader. All the experiments were performed in triplicates.

### Western Blotting

Total protein was lysed using Radio Immunoprecipitation Assay (RIPA) buffer supplemented with protease and phosphatase inhibitor (Beyotime). The supernatant fraction was collected by centrifugation and quantified by BCA assay (Thermo Fisher Scientific). Equal amounts of protein were separated by 10% SDS-PAGE, transferred to PVDF membrane (Millipore, MA, United States), and then blocked with 5% non-fat milk for 1 h. The membrane was probed with the following antibodies: β-catenin (Cat.NO: 8480S, 1:2,000; Cell Signaling Technology, United States), LaminB1(Cat.NO: 13435S, 1:2,000; Cell Signaling Technology, United States) and GADPH (Cat.NO: 5174S, 1:2,000; Cell Signaling Technology, United States) for overnight, then incubated with the appropriate secondary antibody (Merck Millipore, United States) conjugated with HRP (1:2,000 dilution) for 1 h. After washing, the chemiluminescence (Merck Millipore, United States) was used to visualize the bands and GAPDH was used as the internal control.

### Immunofluorescence and Immunohistofluorescence

For immunofluorescence assays, HepG2 and Bel-7402 cells were seeded in 12-well plates and treated with GA for 48 h. Then, cells were fixed with 4% paraformaldehyde for 15 min, 0.5%Triton X-100 was used for permeabilization, and 10% normal donkey serum (Invitrogen) was added and incubated for 1 h. Cells were incubated with primary antibody against β-catenin (Cat.NO. 8480S, 1:100; Cell Signaling Technology, United States) at 4°C for overnight and then probed with donkey anti-rabbit IgG-Alexa Fluor 594 (Absin, Beijing, China) in dark at 37°C for 1 h. Finally, the cells were washed with PBS and stained with DAPI (Beyotime). The images were captured by using a confocal microscope (Nikon, Tokyo, Japan). For immunohistofluorescence examination, the specimens were fixed in 4% paraformaldehyde, dehydrated and embedded in paraffin. Sections (5 µm) were used to analyze Ki-67 and β-catenin expression. After being counterstained with DAPI, the images were captured using a Zeiss Axiophot two microscope.

### Xenograft Animal Model

Twenty-four male BALB/c nude mice (aged 4 weeks old) were purchased from Laboratory Animal Services Centre, Southern Medical University (SMU). The usage and treatment of animals were approved by Institutional Animal Care and Use Committee (IACUC) of SMU (Guangzhou, China, Approval No. L2018187). HepG2 cells (1 × 10^6^) were subcutaneously injected into the dorsal flank of the nude BALB/c mice. When the tumor volume could be palpable, animals were randomly assigned to four groups (*n* = 6). Group 1 (Contro1) was intragastrically administrated with normal saline; Group 2 (GA *ig*) was intragastrically administrated with GA (80 mg/kg); Group 3 (Control 2) was intraperitoneally injected with normal saline; Group 4 (GA *ip*) was intraperitoneally injected with GA (80 mg/kg). The treatment was performed every day for 4 weeks, and tumor size was measured twice a week. The tumor volumes (V) were calculated with the formula: V = *½*×S^2^×L, where S and L are the shortest and longest diameters of the tumor, respectively. At the end of experiments, mice were sacrificed and the tumors were dissected for further investigation.

### TUNEL Staining

For detection of apoptotic cells in tumor tissues, a DeadEnd™ Fluorometric TUNEL System (Promega, China) was used in this study. According to the manufacturer’s instructions, nucleus was counterstained with DAPI, and the images were captured using a confocal microscope (ZEISS, Germany).

### Immunohistochemistry

The tumor sections were used to examine the expression of E-cadhein (Cat.NO: ab231303, Abcam), Vimentin (Cat.NO: ab92547, Abcam) and MMP9 (Cat.NO: ab76003, Abcam) with 1:50 dilution. Visualization was achieved by using the 3, 3′-diaminobenzidine substrate (Dako, Denmark) followed by counterstaining with hematoxylin. The representative images were taken with ×40 magnification and the positive cells were quantified with ImageJ.

### Statistical Analysis

Data were reported as mean ± SEM from at least three independent experiments. Data were analyzed by two-tailed unpaired Student’s t test between two groups and by one-way ANOVA followed by the Dunnett post hoc test for multiple comparison. Statistical analysis was carried out using Graphpad prism software version 8. A *p*-value of <0.05 was considered to be statistically significant. Data are presented as means ± SEM from three independent experiments. *p < 0.05, **p < 0.01,***p < 0.001.

## Results

### GA Significantly Suppressed Cell Viability in HCC Cells

**FIGURE 1:**
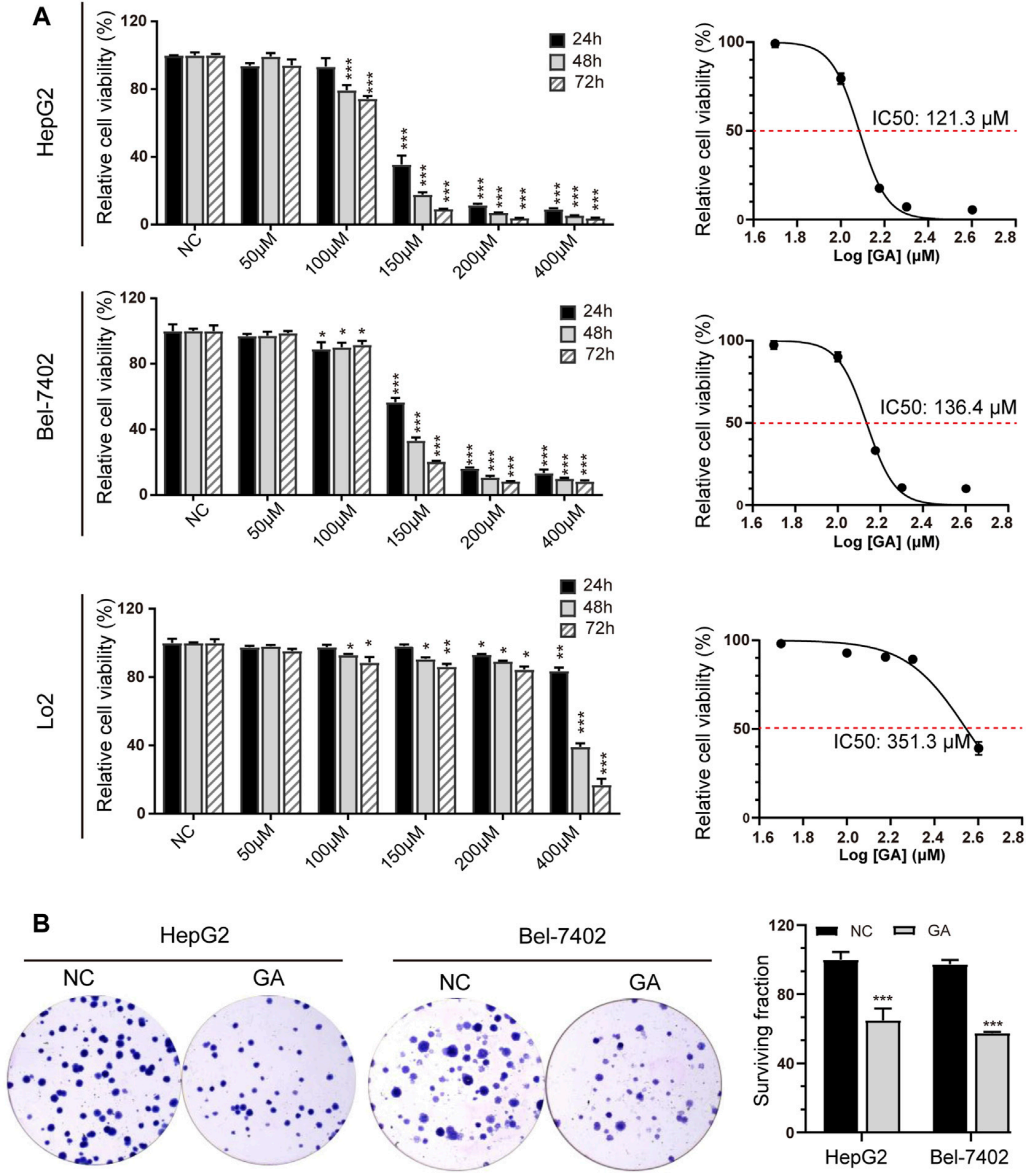
GA significantly suppressed cell viability in HCC cells. **(A)** HepG2, Bel-7402 and LO2 cells were treated with various concentrations of GA (0, 50, 100, 150, 200 and 400 μM), and cell viabilities were measured by CCK-8 assays and the IC50 values were calculated and presented. The data was shown as mean ± SEM (*n* = 5). **(B)** HepG2 and Bel-7402 cells were treated with 120 μM GA for 48 h and subsequently re-plated into 6-well plates and cultured for 2 weeks to assess clonogenic ability. *, *p* < 0.05; **, *p* < 0.01; ***, *p* < 0.001; *vs* NC.

In order to determine the anti-cancer activity of GA, HepG2 and Bel-7402 cells were treated with various concentration of GA. The cell viability was measured and GA was found to significantly inhibit the cell viability in a concentration-dependent manner (Figure 1A). In addition, we also found that it had a weak effect on the cell growth of LO2, the immortalized non-tumorigenic human hepatocyte cells. The IC50 value of GA was 121.3 μM for a 48 h treatment in HepG2 cells and it was 136.4 μM in Bel-7402 cells. So we selected the concentration of 120 μM for further investigation. The colony formation assays showed that fewer and smaller colonies were observed in GA treated HepG2 and Bel-7402 cells when compared with negative control group (Figure 1B).

### GA Induced Cell Cycle Arrest and Apoptosis in HCC Cells

**FIGURE 2:**
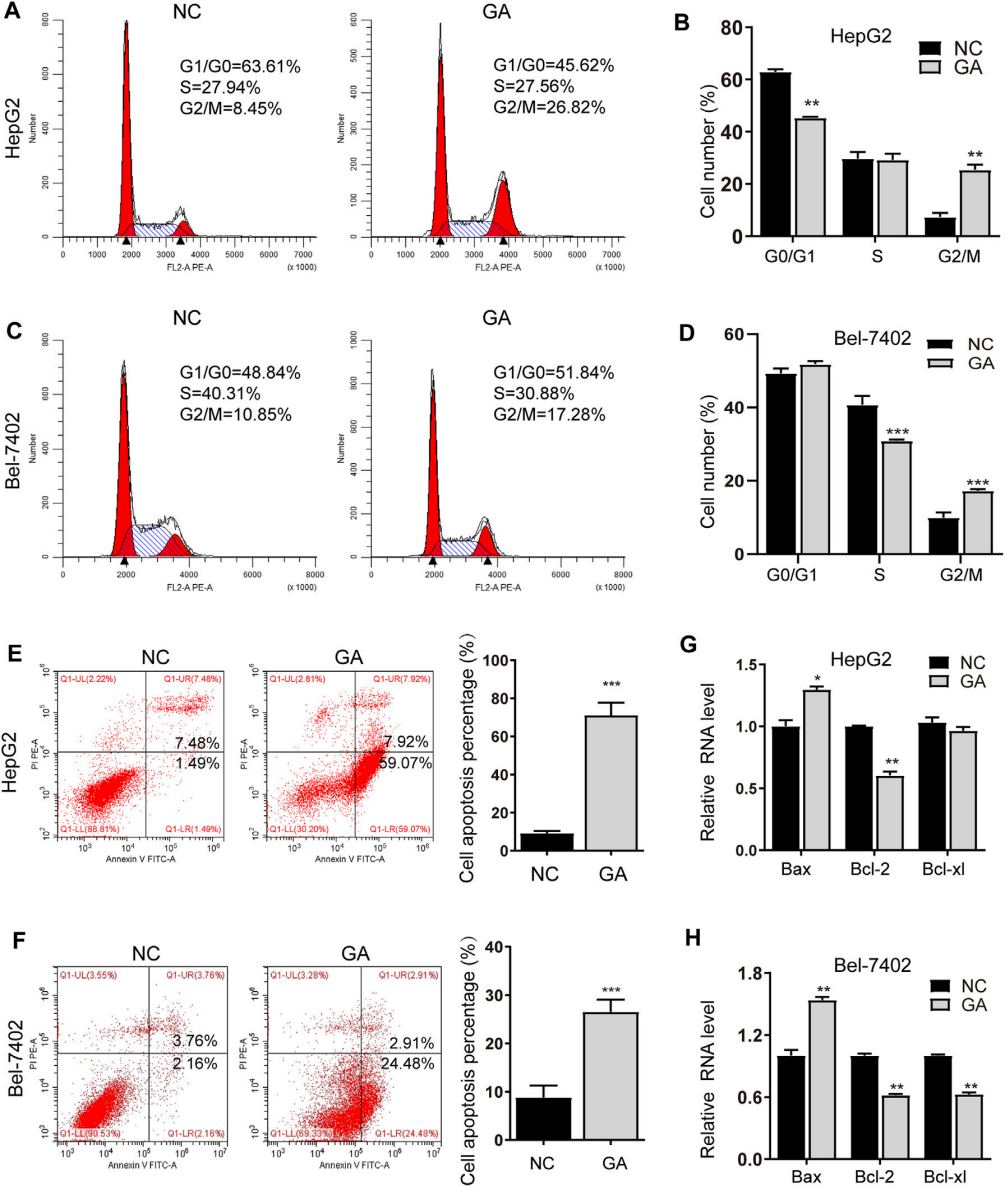
GA induced cell cycle arrest and apoptosis in HCC cells. HepG2 and Bel-7402 cells were treated with 120 μM GA for 48 h, and cell cycles were determined by flow cytometry assay. GA induced the significant arrest at G2/M phase in HepG2 cell **(A,B)** and Bel-7402 cell **(C,D)**. GA induced apoptosis in HCC cells. **(E,F)** HepG2 **(E)** and Bel-7402 **(F)** cells were tested with 120 μM GA for 48h, and the apoptotic cells were examined by annexin V-FITC and PI double staining. **(G,H)** The mRNA expression of Bax, Bcl-2 and Bcl-xl were examined in GA-treated HCC cells. GAPDH were used as an endogenous control. *, *p* < 0.05; **, *p* < 0.01; ***, *p* < 0.001; *vs* NC.

To elucidate the underlying anti-cancer mechanisms of GA, the GA-treated HepG2 and Bel-7402 cells were subjected to cell cycle analyses. The cell cycle distribution demonstrated that GA induced an increased percentage of cells in G2/M phase for HepG2 cells and Bel-7402 cells (Figures 2A–D). In the following investigation, HepG2 and Bel-7402 cells were treated with GA and quantitative analyses of the apoptotic cells were examined. As shown in Figures 2E,F, 52.3 and 27.4% apoptotic cells were observed in GA-treated HepG2 cells and Bel-7402 cells respectively. Bcl-2 family members including the pro-apoptotic regulator Bax and the anti-apoptotic regulators Bcl-2 and Bcl-xl were also examined to further validate the apoptosis induced by GA. As shown in Figures 2G,H, Bax was up-regulated by GA in the two HCC cells. Moreover, both Bcl-2 and Bcl-xl were all down-regulated by GA in two HCC cells, while Bcl-xl expression was slightly decreased in HepG2 cells (Figure 2G).

### GA Inhibited the EMT and Metastasis of HCC Cells

**FIGURE 3:**
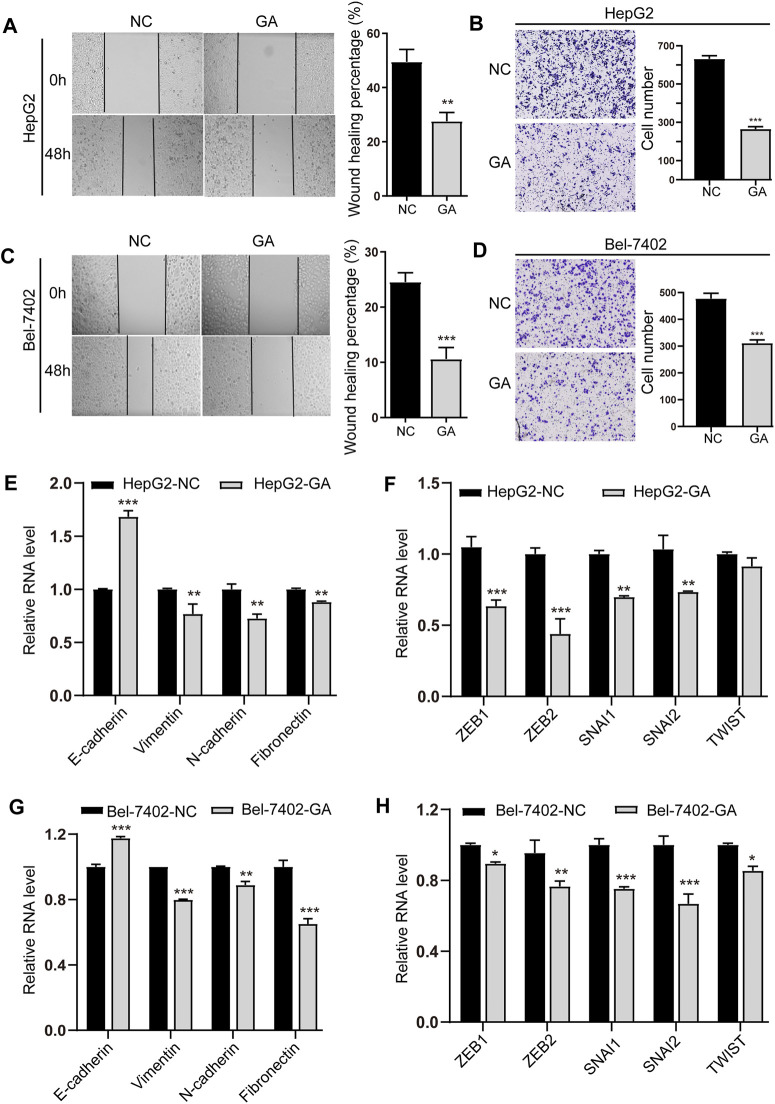
GA inhibited the EMT and metastasis of HCC cells. HepG2 **(A)** and Bel-7402 **(C)** cells were treated with 80 μM for 48 h, and wound-healing migration was assayed. The HepG2 **(B)** and Bel-7402 **(D)** cells treated with 80 μM GA and invasion was examined. **(E,G)** The mRNA expression of E-cadherin, Vimentin, N-cadherin and Fibronectin in GA-treated HepG2 cells **(E)** and Bel-7402 cells **(G)** were measured by q-RT-PCR. **(F,H)** The mRNA expression of ZEB1, ZEB2, SNAI1, SNAI2, TWIST in GA-treated HepG2 cells **(F)** and Bel-7402 cells **(H)** were measured by q-RT-PCR. Data are presented as mean ± SEM. *, *p* < 0.05; **, *p* < 0.01; ***, *p* < 0.001; *vs* NC.

We next investigated the suppressive effects of GA on invasion and metastasis in HCC cells. A concentration below IC50, 80 μM was chosen for the invasion and metastasis examination to exclude the cell proliferation’s impact. The results of wound healing migration showed that the bigger scratch area was observed in GA treated cells (Figures 3A–C), suggesting the suppressive effect of GA on migration in HCC cells. The transwell assays also exhibited less invaded cells in GA-treated cells than non-treated groups (Figures 3B–D), which confirmed the wound healing results.

Moreover, the epithelial marker E-cadherin was found to be increased, while the mesenchymal marker genes like Vimentin, N-cadherin and Fibronectin were decreased in GA-treated cells (Figures 3E–G). Consistent with the decreased expression of EMT-inducing transcription factors (EMT-TFs) like ZEB1, ZEB2, SNAI1, SNAI2 and TWIST (Figures 3F–H), suggesting that GA may regulate EMT and metastasis of HCC cells.

### GA Suppressed the Wnt/β-Catenin Signaling and the Expression of MALAT1 in HCC Cells

**FIGURE 4:**
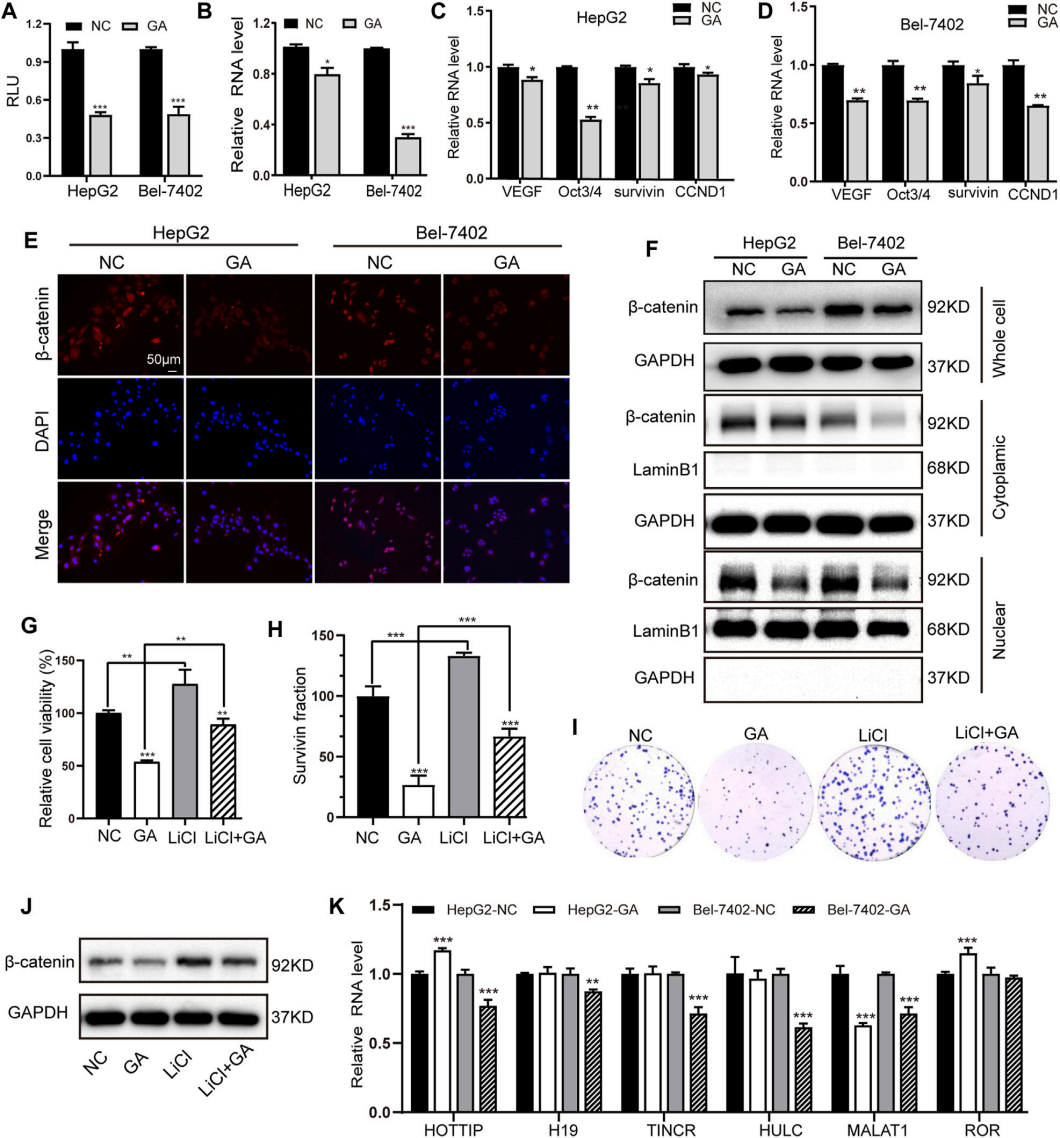
GA induced the inactivation of Wnt/β-catenin signaling and depression of MALAT in HCC cells. **(A)** After transfection with TOPflash luciferase reporter, the two HCC cells were treated with 120 μM GA for 48 h. The luciferase activities were measured. **(B)** The mRNA expression of β-catenin in GA-treated cells were examined by q-RT-PCR. **(C,D)** The expression of several downstream targets of Wnt/β-catenin pathway were examined by qPCR assays. **(E)** The expression of β-catenin was detected by immunofluorescence staining. **(F)** The protein level of total β-catenin, intracytoplasmic β-catenin and intranuclear β-catenin in GA-treated cells were determined by Western blotting. Cell viabilities **(G)** and colony formation **(H,I)** was measured after treatment of LiCl and GA. **(J)** The protein level of total β-catenin were determined by Western blotting after treatment of LiCl and GA. **(k)** The two HCC cell lines were incubated with GA for 48 h, RNA expression of chosen lncRNAs were determined by qRT-PCR assays. *, *p* < 0.05; **, *p* < 0.01; ***, *p* < 0.001; *vs* NC.

The canonical Wnt/β-catenin signaling has been demonstrated to play crucial regulatory roles in the development of tumorigenesis in HCC (Clevers, 2006). We therefore investigated whether this signaling could be involved in the GA-mediated anti-cancer activity. The Wnt/β-catenin signaling reporter TOPflash was transfected in the GA-treated HepG2 and Bel-7402 cells, and the luciferase activity was examined. It was showed that GA impaired the luciferase activity of TOPflash in HCC cells (Figure 4A). Moreover, the expression of β-catenin was suppressed by GA at mRNA level (Figure 4B) and several downstream target genes of Wnt/β-catenin signaling such as VEGF, Oct3/4, survivin, CCND1 were down-regulated in GA treated cells compared with negative control cells (Figures 4C,D). Furthermore, We analyzed the protein level of total β-catenin, intracytoplasmic β-catenin and intranuclear β-catenin and found that the decreased expression of total and nuclear β-catenin in the GA-treated HCC cells (Figure 4F). The further immunofluorescence analyses demonstrated that the decreased expression of β-catenin was observed in GA-treated HCC cells (Figure 4E).

To further confirm whether Wnt/β-catenin signaling could be involved in the GA-mediated anti-cancer activity in HCC, LiCl, which has been demonstrated to stimulate Wnt/β-catenin signaling in our previous study (Lv et al., 2019) was applied in the present study. HepG2 cells were pre-treated with 20 mM LiCl for 1h, and then followed with GA treatment for the anti-cancer effect examination. The results of cell viability and colony formation demonstrated that LiCl successfully reversed the anti-cancer effect of GA on HCC cells (Figures 4G–I). Besides, the suppressive expression of total β-catenin induced by GA was partially reversed by LiCl stimulation (Figure 4J). All of these data indicated that GA induced the inactivation of Wnt/β-catenin signaling in HCC cells. Many lncRNAs have demonstrated to be severed as critical regulators in the Wnt/β-catenin signaling pathway in HCC (Wong et al., 2018). To further identify the putative lncRNAs involved in this process, six previous reported lncRNAs including HOTTIP, H19, TINCR, HULC, MALAT1 and ROR were chosen and subjected for qRT-PCR examination to check their expression profiling. Among these candidates, only MALAT1 presented significant down-regulation in two HCC cells (Figure 4K).

### MALAT1 Mediated the Suppressive Effects of GA on Hepatocellular Carcinoma Through Inactivating Wnt/β-Catenin Signaling

**FIGURE 5:**
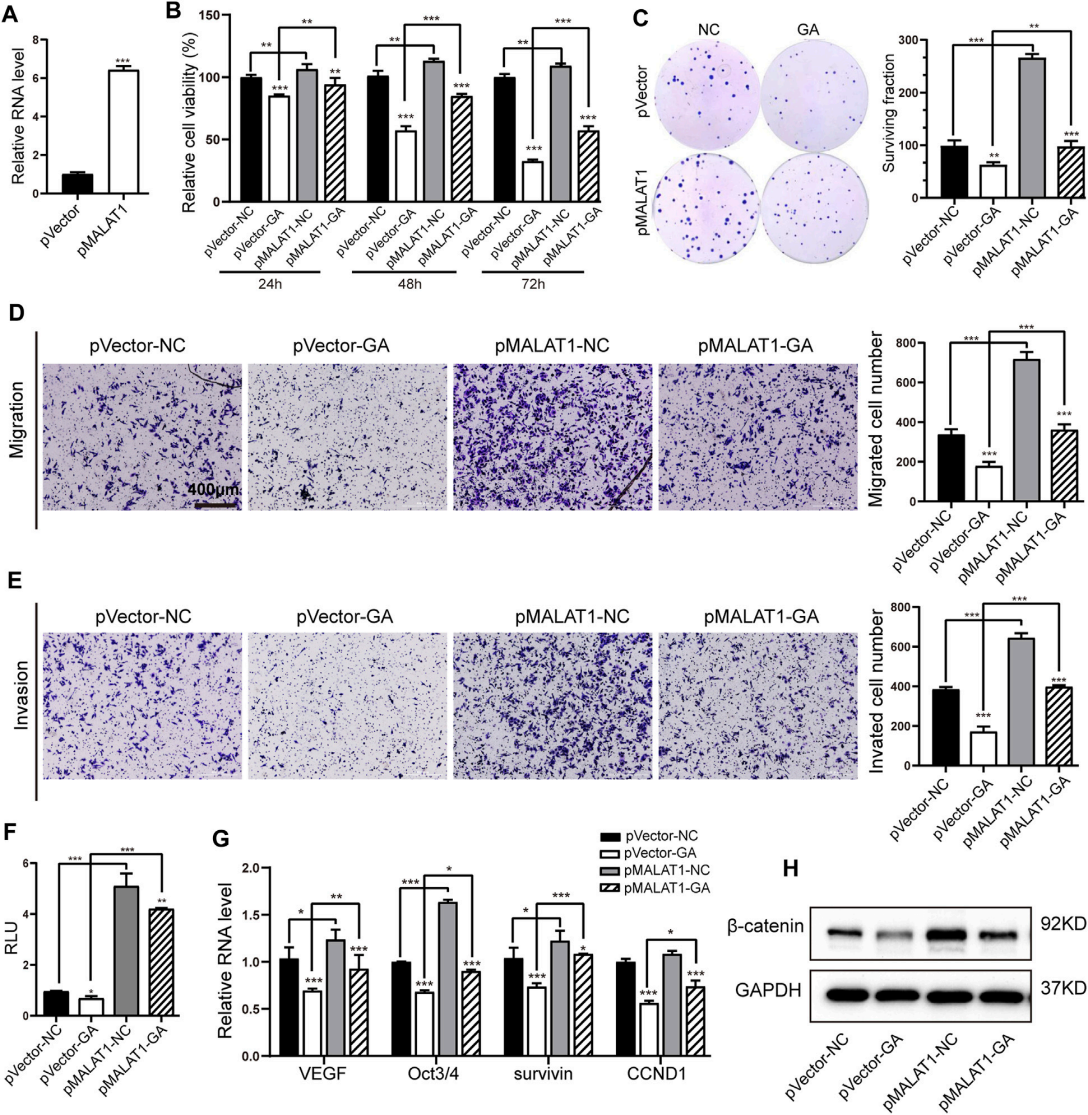
MALAT1 reversed the suppressive effects of GA on HCC and Wnt/β-catenin signaling. **(A)** The expression of MALAT1 in MALAT1-overexpressing HepG2 cells by qRT-PCR examination. **(B,C)** The cell viabilities **(B)** and colony formation **(C)** were measured with GA treatment in MALAT1-overexpressing HCC cells. **(D,E)** Transwell assays for migration **(D)** and invasion **(E)** with GA treatment in MALAT1-overexpressing HepG2 cells. **(F)** The luciferase activities were examined after 48 h treatment of GA in MALAT1 overexpressing HepG2 cells. **(G)** Wnt/β-catenin downstream target genes were examined by qRT-PCR in GA-treated MALAT1-overexpressing HepG2 cells. **(H)** The protein level of total β-catenin was examined with GA treatment in MALAT1 overexpressing HepG2 cells by Western blotting analyses. *, *p* < 0.05; **, *p* < 0.01; ***, *p* < 0.001; vs NC.

MALAT1 has been reported to mediate the proliferation and metastasis in HCC through Wnt/β-catenin pathway (Chang et al., 2020). So we wondered whether this lncRNA-mediated Wnt signaling participated in the GA-induced anti-cancer activity. A MALAT1-overexpressing HepG2 cell line was generated, and subsequently qRT-PCR examination showed that MALAT1 was obviously up-regulated in this cell line (Figure 5A). The examination of cell viability and colony formation showed that MALAT1 overexpression partially reversed the GA-induced cell proliferative inhibition (Figures 5B,C). The further transwell assays demonstrated that MALAT1 successfully reversed the suppressive effect of GA on metastasis in HCC cells (Figures 5D,E). As for the Wnt/β-catenin signaling, our results revealed that MALAT1 reinforced overexpression significantly abolished the suppressive effect of GA on the luciferase activity of Wnt signaling reporter TOPflash (Figure 5F), and reversed the down-regulation of total β-catenin and several downstream target genes of Wnt/β-catenin signaling (Figures 5G,H).

### GA Suppressed Tumor Growth in Xenograft Animal Model

**FIGURE 6:**
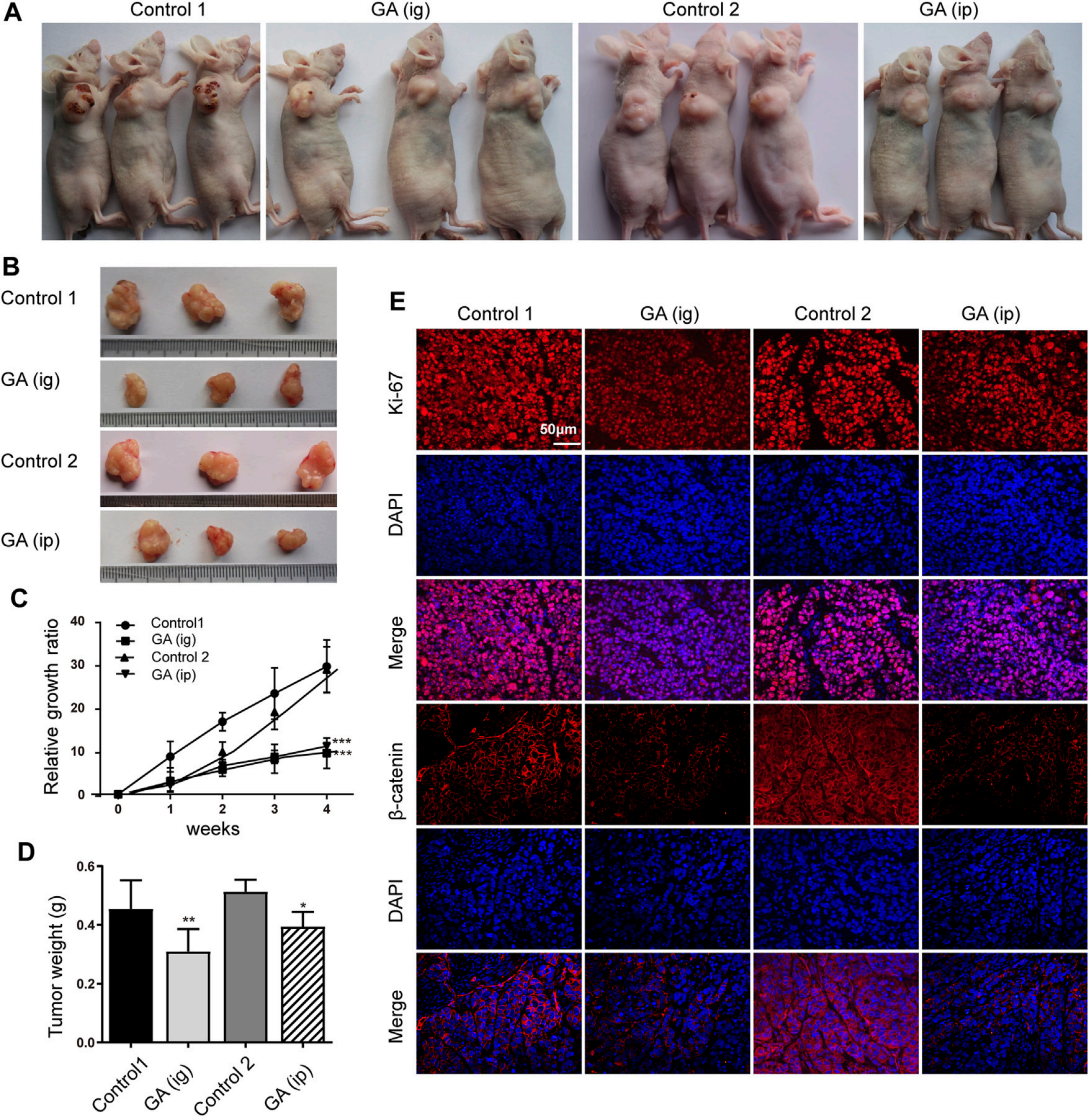
GA reduced the tumor growth of HCC cells *in vivo.* HepG2 cells were subcutaneously injected into the dorsal flank of nude mice, and GA was administrated *i. g* or *i. p*. **(A,B)** The representative images showed smaller tumors in GA treated groups when compared with control groups. **(C)** The growth curve of tumor volumes. **(D)** The tumor weight. **(E)** The immunofluorescence of Ki-67 and β-catenin stained sections followed by counterstaining with DAPI. Each data represented the mean ± SEM of six mice. *, *p* < 0.05; **, *p* < 0.01; ***, *p* < 0.001; *vs* control1 or control 2.

**FIGURE 7:**
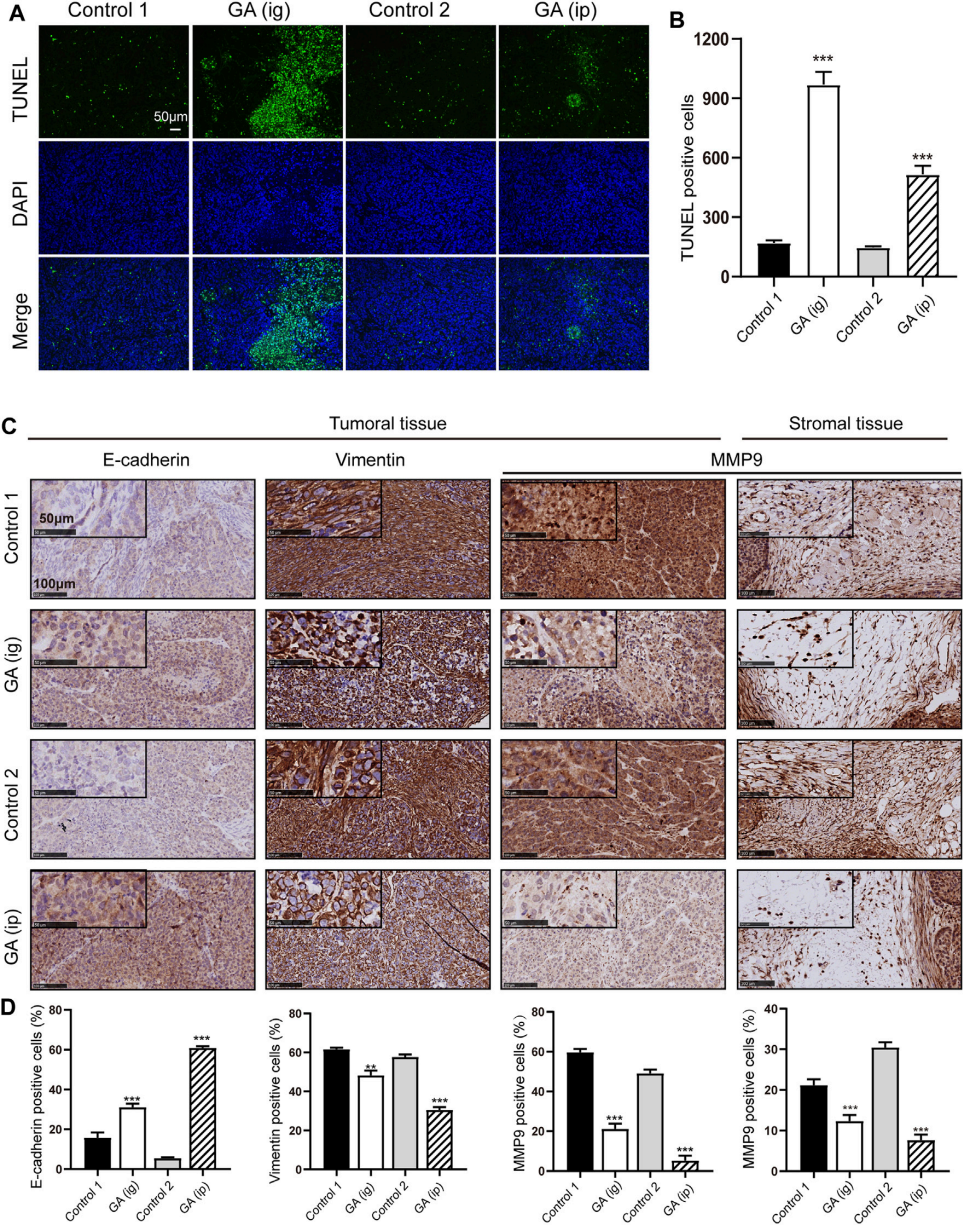
GA induced the apoptosis and inhibited metastasis *in vivo.* **(A)** the apoptotic cells in tumor specimens were assessed by using TUNEL assays. **(B)** Quantitative analyses of TUNEL-positive cells. **(C)** IHC detection of E-cadherin, Vimentin and MMP9 in tumoral or stromal tissue. **(D)** Quantitative analyses of IHC examination. *, *p* < 0.05; **, *p* < 0.01; ***, *p* < 0.001; *vs* Control one or control 2.

We next examined the *in vivo* function of GA in the tumorigenesis using an xenograft model. After that HepG2 cells were subcutaneously injected into the nude mice, GA were administered intraperitoneally (*ip*) and intragastrically (*ig*) every day. Strikingly, we found that the control group carried larger burden when compared with treated groups (Figures 6A,B). Furthermore, a significant reduction in tumor growth (Figure 6C) and weight (Figure 6D) was also observed in GA-treated groups. To better elucidate the underlying mechanism of GA, the expressions of Ki-67 and β-catenin in tumor tissues were examined by immunohistofluorescence analyses. Decreased Ki-67 and β-catenin expression were exhibited in GA treated groups (Figure 6E). Moreover, the apoptotic cells in tumor tissues were monitored, and a significant increase of apoptotic cells was observed GA in GA-treated tissues (Figures 7A,B). On the other hand, the metastasis related genes were examined by IHC assays, and it was found that E-cadherin was up-regulated; Vimentin and MMP9 were down-regulated in GA-treated tumoral or stromal tissue (Figures 7C,D), suggesting that GA inhibited the EMT polarization and blocked the potential of metastatic dissemination of primary carcinoma cells *in vivo*.

## Discussion

Gallic acid is a kind of phenolic acids that can be widely found in dietary substances and traditional medicine herbs. Its anti-cancer activities has been reported in various cancers such as leukemia (Chen and Chang, 2012; Batista and Chang, 2013; Locatelli et al., 2013), melanoma (Su et al., 2013), prostate cancer (Russell et al., 2012; Heidarian et al., 2016), lung cancer (Park and Kim, 2013), gastric cancer (Ho et al., 2010), colon cancer (Forester et al., 2014) and oral cancer (Kuo et al., 2014). However, the anti-cancer effect on liver cancer remains unclear and the detailed mechanisms have not been clarified systematically. Therefore, depicting the detailed mechanisms of GA suppressed the tumorigenesis in HCC could provide the strong basis for its clinical practice in the near future.

To investigate the *in vitro* function of GA in HCC, cell viability was examined by CCK-8 assays. The results revealed that GA suppressed cell viability in a time and concentration-dependent manner in HCC cells, while exhibited a weak effect on the immortalized normal liver cell line LO2, suggesting non-obvious cytotoxicity *in vitro*.

As previously reported, GA exhibited no adverse action or health-related acute or sub-acute toxicity of GA in albino mice (Variya et al., 2019). Furthermore, it also has the hepatoprotective activity (Anand et al., 1997). Our further *in vitro* investigation showed that GA induced an increased percentage of cells in G2/M phase for HepG2 cells and Bel-7402 cells. As well known, the G2/M checkpoint prevents cells from initiating mitosis in the presence of DNA damage, once the cellular DNA damage is too extensive to be repaired, apoptosis pathways come into operation to eliminate the damaged cells. Moreover, GA was also found to induce the apoptosis in HCC cells, which was consistent with the previous studies (Lima et al., 2016; Sun et al., 2016). We also found that the pro-apoptotic regulator Bax was up-regulated, and the anti-apoptotic regulators Bcl-2 and Bcl-xl were down-regulated by GA, while Bcl-xl expression was slightly decreased in HepG2 cells. Among the expression of these regulators, most of them validated the apoptotic effect of GA. The TUNEL assays also demonstrated that GA induced the *in vivo* apoptosis in tumor specimens. It has been reported that, GA with low concentrations has an antioxidant potential that can prevent oxidative damage to cellular DNA (Freiría-Gándara et al., 2018; Kosuru et al., 2018); however, at higher concentrations, it may induce cellular DNA damage and late cell cycle arrest, and also initiates apoptosis in multiple cancers (Liu et al., 2013; Weng et al., 2015).

As for the effects of GA on migration, there are several reports to document that GA inhibited the migration and motility in various cancer cells including leukemia, non-small lung cancer, gastric cancer and oral cancer (Ho et al., 2010; Chen and Chang, 2012; Kuo et al., 2014; Sunil Gowda et al., 2018). As well known, most cancers originate from epithelial cells, and the epithelial-mesenchymal transition (EMT) converts epithelial cells into mesenchymal cells which contribute to tumor progression and metastasis (Thiery et al., 2009). Regulated by microenvironment signals, this cellular plasticity process is driven by a network of embryonic EMT-inducing transcription factors (EMT-TFs) mainly represented by the SNAIL, TWIST, and ZEB protein families, which interact with epigenetic regulators (De Craene and Berx, 2013; Tam and Weinberg, 2013). The up-regulation of E-cadherin and down-regulation of Vimentin, N-cadherin, Fibronectin and some EMT-TFs like ZEB1, ZEB2, SNAI1, SNAI2 and TWIST were observed in GA-treated HCC cells. The activation of EMT programmes orchestrated by EMT- TFs in primary carcinoma cells is a critical and initial step in the metastatic dissemination of various types of carcinoma cells. Besides, MMP-9 is a crucial enzyme of MMPs which regulates the degradation of the main constituent of the extracellular matrix (ECM) and is deeply involved in cancer invasion and metastasis (Stamenkovic, 2000; Kessenbrock et al., 2010). In our present study, the up-regulation of E-cadherin and down-regulation of Vimentin and MMP9 were found in GA-treated tumors, combined with the decreased expression of MMP9 in tumor adjacent stromal tissue, which demonstrated that GA inhibited the EMT polarization and blocked the potential of metastatic dissemination of primary carcinoma cells *in vivo*. Our *in vitro* and *in vivo* results firstly demonstrated that GA significantly suppressed the EMT polarization and metastasis of HCC cells. Therefore, our data combined with the previous reports suggest that GA may be a promising anti-cancer candidate and potentially be developed as a therapeutic drug for clinical practice in the near future.

As well known, Wnt/β-catenin signaling play critical roles in many physiological processes and disease progression. Mostly, it is inactive in the mature liver but becomes re-activated in certain pathological conditions such as cancer. Incomplete statistics, 20–35% HCC cases show aberrant activation of this pathway (Russell and Monga, 2018; Perugorria et al., 2019). As the Wnt ligands bind to frizzled receptors, β-catenin, the core regulator of this signaling cascade is translocated from cytoplasm towards the nucleus, and subsequently initiates the transcription of target genes (Russell and Monga, 2018; Liang et al., 2019; Lv et al., 2019). Therefore, targeting Wnt/β-catenin signaling could provide insight for developing the new therapeutic interventions for cancer patients. Gallic acid has been reported to promote osteoblast differentiation by stimulating the P38 MAP kinase (MAPK)/β-catenin canonical Wnt signaling (Chen et al., 2010). Additionally, GA was also demonstrated to suppress the expression of cancer stem cells (CSC) markers as well as the β-catenin/p-GSK3β signaling in colorectal cancer (Lee et al., 2016). Moreover, GA exhibited the inhibitory effects on melanin synthesis by inducing the inactivation of Wnt/β-catenin and cAMP signaling in melanoma cells (Su et al., 2013). We thereby hypothesized that Wnt/β-catenin signaling might get involved in the GA-induced anti-tumorigenesis in HCC, and our results also validated that GA induced the inactivation of Wnt/β-catenin signaling *via* examining the luciferase activity of Wnt signaling reporter and the expression of β-catenin and several downstream target genes of this signaling.

LncRNA have been considered as crucial regulators in tumorigenesis, and dysregulated lncRNAs may serve as therapeutic targets or diagnosis biomarkers for patients (Huarte, 2015; Wong et al., 2018). As we previously described, Wnt/β-catenin signaling is one of the most important pathway in tumorigenesis. We searched the reference database and obtained several lncRNA candidates including HOTTIP, H19, MALAT1, TINCR, HULC and linc-ROR. These lincRNAs have been demonstrated to exert their function in tumor biology through activating Wnt/β-catenin signaling. HOTTIP could maintain the stemness of cancer stem cells (PCSCs) through the HOTTIP/WDR5/HOXA9/Wnt axis in pancreatic cancer (Fu et al., 2017); lncRNA H19 mediated the methotrexate resistance in colorectal cancer through activating Wnt/β-catenin signaling (Wu et al., 2017). The overexpression of MALAT1 increased the proliferation and metastasis though MALAT1/Wnt signaling axis in HCC (Chang et al., 2020). The lncRNA PLAC2 (also known as TINCR) is transcriptionally activated by H3K27ac modification at the promoter region in oral squamous cell carcinoma (OSCC), and promotes cell growth and metastasis *via* activating Wnt/β-catenin signaling pathway (Chen et al., 2019). HULC could activate PI3K/AKT and Wnt/β-catenin pathways activities in thyroid cancer (Yang et al., 2020). Moreover, linc-ROR induces EMT in ovarian cancer cells by increasing Wnt/β-catenin signaling (Lou et al., 2017). In this study, these lncRNAs were chosen for examination of their expression profiling. Of which, MALAT1 was down-regulated in two GA-treated HCC cells. MALAT1 may act as a potential therapeutic target and molecular biomarker in HCC, which regulates the proliferation, metastasis, cancer cell metabolism as well as the stemness of Hepatic CSCs *via* multiple mechanisms. Furthermore, MALAT1 overexpression partially reversed the GA-induced the inhibitory proliferation and metastasis; and successfully abolished the suppressive effects on Wnt/β-catenin signaling. Taken together, targeting the MALAT1-Wnt/β-catenin signaling regulatory axis was the underlying mechanisms of GA suppressed tumorigenesis. To sum up, our data demonstrated that GA suppressed tumorigenesis through an lncRNA MALAT1-Wnt/β-catenin axis in HCC. These information gained from this study provides a new insight into cancer prevention as well as developing a novel synergistic intervene between GA and traditional chemotherapy agents.

## Data Availability

The original contributions presented in the study are included in the article/Supplementary Material, further inquiries can be directed to the corresponding author/s.

## Abbreviations

CCK8, cell counting assay kit-8; CCND1, cyclin D1; DAPI, 4′,6-diamidino-2-phenylindole; EMT, epithelial to mesenchymal transition; GA, gallic acid; GADPH, glyceraldehyde 3-phosphate dehydrogenase; HCC, hepatocellular carcinoma; lncRNA, long noncoding RNA; MALAT1, metastasis-associated lung adenocarcinoma transcript 1; MMP9, matrix metalloproteinase 9; NC, negative control；PI, propidium iodide; qRT-PCR, quantitative real-time polymerase chain reaction; SEM, standard error of the mean; VEGF, vascular endothelial growth factor.
