# Supplementary material for: Gallic Acid Suppressed Tumorigenesis by an LncRNA MALAT1-Wnt/β-Catenin Axis in Hepatocellular Carcinoma
Source: Front Pharmacol. 2021 Oct 6;12:708967. doi: 10.3389/fphar.2021.708967 (PMC8526893; doi:10.3389/fphar.2021.708967)
Supplement: Supplementary file 1 [file DataSheet4.docx]

The original images and data are in folders were labeled with each figure and upload to <https://www.jianguoyun.com/>

Figure 1 <https://www.jianguoyun.com/p/DWWy4N0QrOzHCRja6_gD>

Figure 2 <https://www.jianguoyun.com/p/DfZkTVoQrOzHCRik6_gD>

Figure 3 <https://www.jianguoyun.com/p/DVK9F6oQrOzHCRjg6_gD>

Figure 4 <https://www.jianguoyun.com/p/DamoGiAQrOzHCRjh6_gD>

Figure 5 <https://www.jianguoyun.com/p/DWPnJ0sQrOzHCRji6_gD>

Figure 6 <https://www.jianguoyun.com/p/DVesAigQrOzHCRjk6_gD>

Figure 7 <https://www.jianguoyun.com/p/DUJpoVcQrOzHCRjm6_gD>
