# Supplementary figures and images for: Gallic Acid Suppressed Tumorigenesis by an LncRNA MALAT1-Wnt/β-Catenin Axis in Hepatocellular Carcinoma
Source: Front Pharmacol. 2021 Oct 6;12:708967. doi: 10.3389/fphar.2021.708967 (PMC8526893; doi:10.3389/fphar.2021.708967)

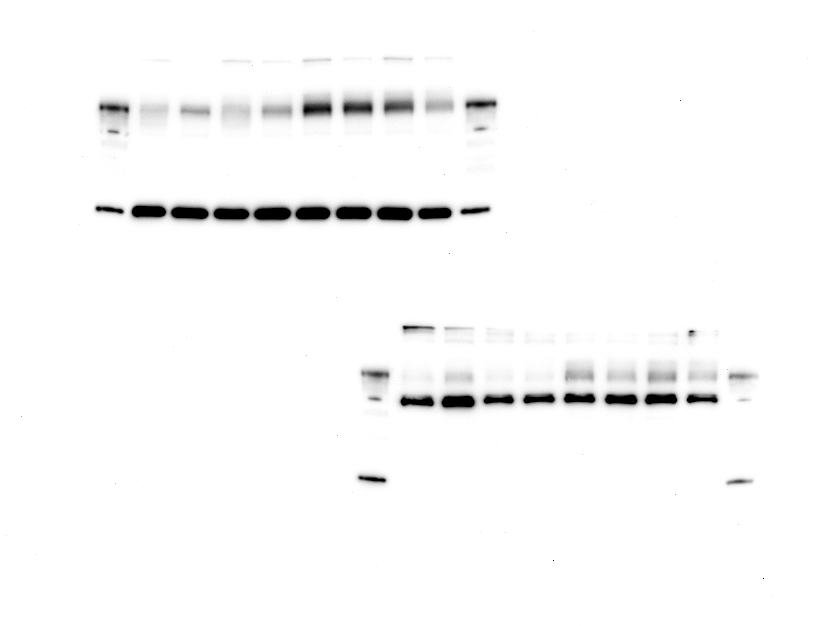

Supplement: Supplementary file 2 [file DataSheet1.ZIP › Western blot scans/Figure 4F-cytoplamic-a┬catenin-LaminB1-GAPDH.tif]

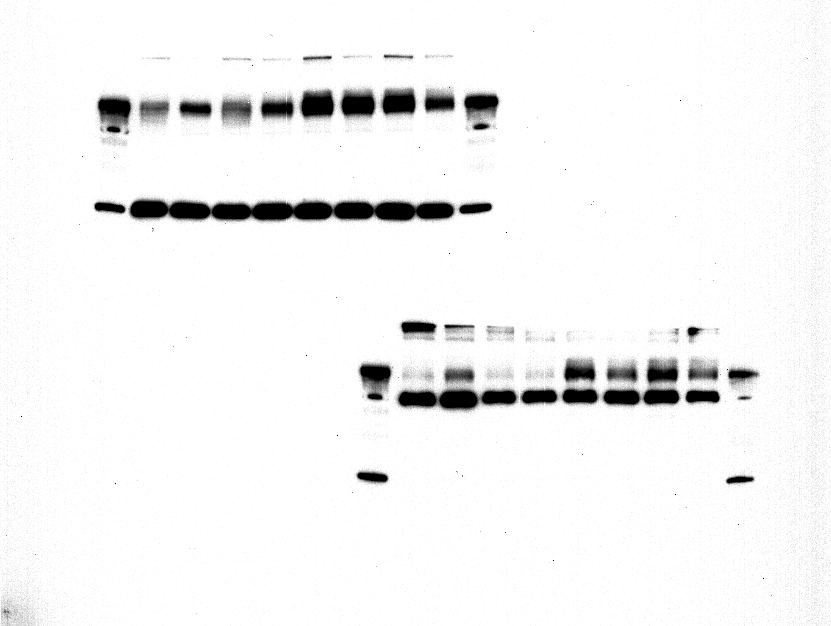

Supplement: Supplementary file 2 [file DataSheet1.ZIP › Western blot scans/Figure 4F-Nuclear-a┬-catenin-LaminB1-GAPDH.tif]

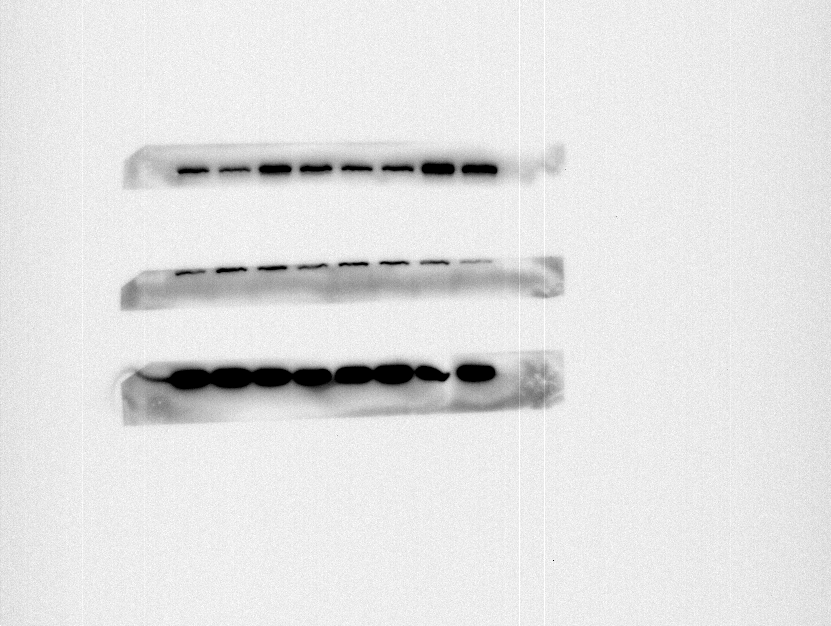

Supplement: Supplementary file 2 [file DataSheet1.ZIP › Western blot scans/Figure 4F-Total a┬-catenin.tif]

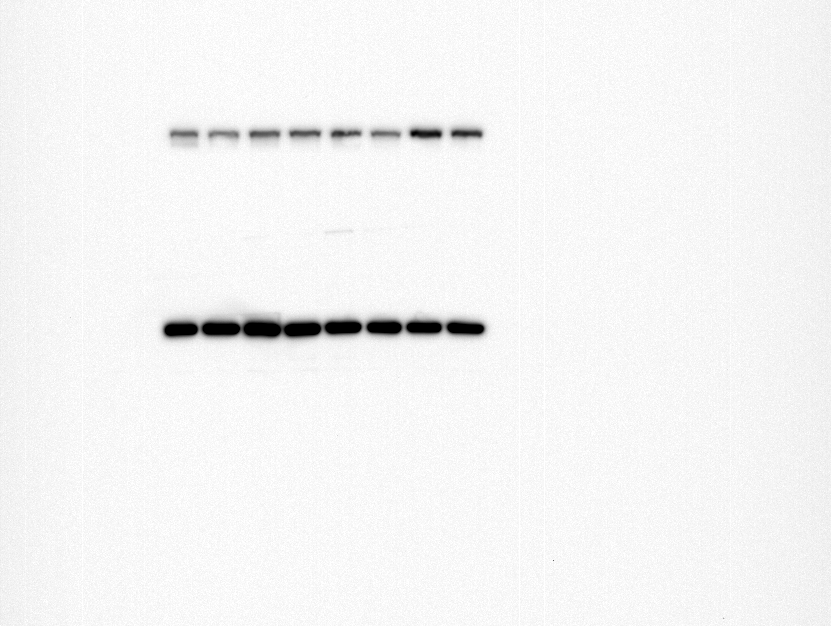

Supplement: Supplementary file 2 [file DataSheet1.ZIP › Western blot scans/Figure 4J-a┬-catenin-GAPDH.png]

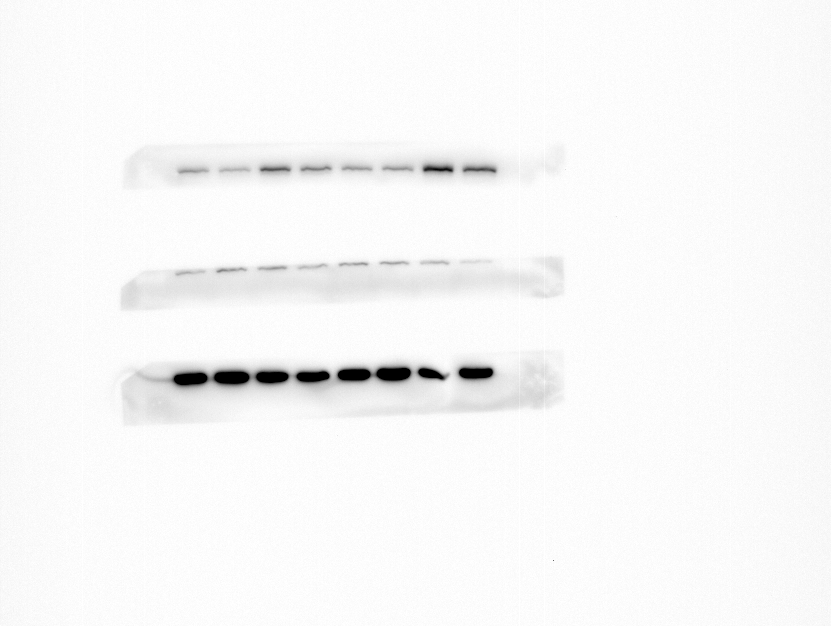

Supplement: Supplementary file 2 [file DataSheet1.ZIP › Western blot scans/Figure 4-total-GAPDH.tif]

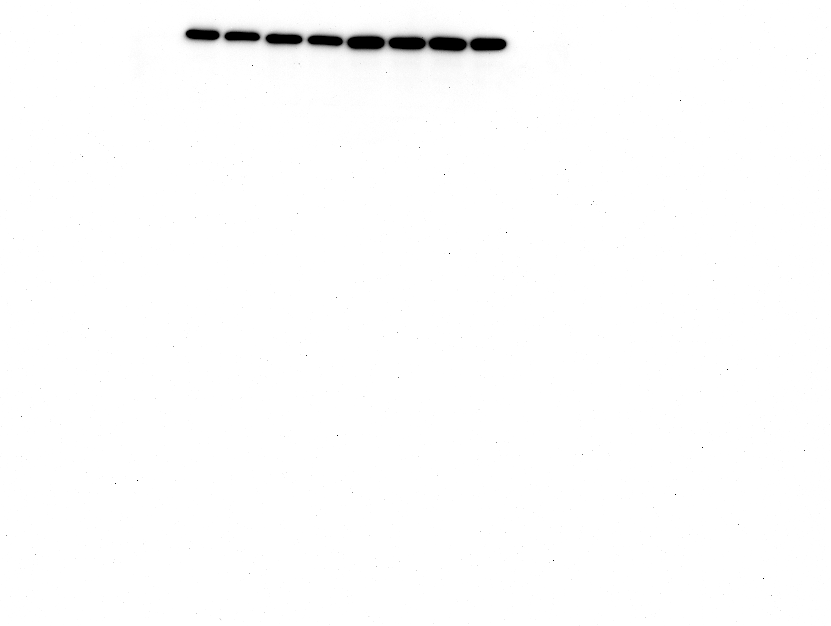

Supplement: Supplementary file 2 [file DataSheet1.ZIP › Western blot scans/Figure 5H-GAPDH.png]

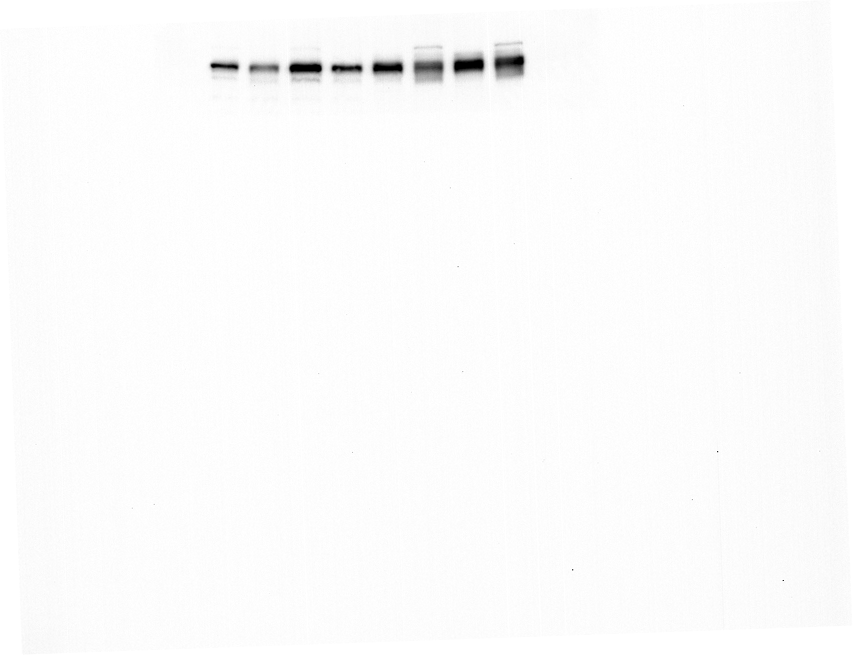

Supplement: Supplementary file 2 [file DataSheet1.ZIP › Western blot scans/Figure 5H-a┬-catenin.png]
